# Correction: Regulation of Cigarette Smoke (CS)-Induced Autophagy by Nrf2

**DOI:** 10.1371/annotation/d13c3d06-8eb8-49ec-8326-2db7487a7a8a

**Published:** 2014-01-06

**Authors:** Lingxiang Zhu, Erika C. Barrett, Yuxue Xu, Zuguo Liu, Aditya Manoharan, Yin Chen

The name of the second author was spelled incorrectly. The correct name is: Erika C. Barrett. The correct Citation is: Zhu L, Barrett EC, Xu Y, Liu Z, Manoharan A, et al. (2013) Regulation of Cigarette Smoke (CS)-Induced Autophagy by Nrf2. PLoS ONE 8(4): e55695. doi:10.1371/journal.pone.0055695. 

